# A highly efficient electrochemical sensor containing polyaniline/cerium oxide nanocomposites for hydrogen peroxide detection

**DOI:** 10.1039/d2ra05041b

**Published:** 2022-11-03

**Authors:** Mahmoud A. Hussein, Ajahar Khan, Khalid A. Alamry

**Affiliations:** Chemistry Department, Faculty of Science, King Abdulaziz University Jeddah 21589 Saudi Arabia maabdo@kau.edu.sa mahussein74@yahoo.com kaalamri@kau.edu.sa; Department of Food and Nutrition, Bionanocomposite Research Center, Kyung Hee University 26 Kyungheedae-ro, Dongdaemun-gu Seoul 02447 South Korea

## Abstract

An efficient electrochemical sensor containing polyaniline/cerium oxide (PANI/CeO_2_) nanocomposites for the detection of hydrogen peroxide has been fabricated using the traditional *in situ* oxidative polymerization process. PANI/CeO_2_ nanocomposite-based modified glassy carbon electrodes were utilized as an electrochemical sensor for the detection of hydrogen peroxide. Before the fabrication, CeO_2_ was prepared by a hydrothermal method, and common techniques confirmed its structure. PANI/CeO_2_ nanocomposites were prepared by adding variable loadings of the pre-prepared CeO_2_ nanoparticles (weight%) inside the polymer host matrix. All the nanocomposites were characterized to determine their chemical structures and suitability for electrode materials. The electrode detection limit, sensitivity, and effect of pH on the sensor performance were investigated using different electrochemical methods, including cyclic voltammetry, electrochemical impedance spectroscopy, and linear sweep voltammetry. The results indicated that the sensing abilities of the synthesized PANI/CeO_2(10)_ nanocomposite-modified GCE presented good electrocatalytic oxidation properties towards H_2_O_2_ with an enhanced low limit of detection and good repeatability. The fabricated electrode sensor was successfully used to detect H_2_O_2_ in real samples.

## Abstract 2

An efficient electrochemical sensor containing (PANI/CeO_2_) for the detection of hydrogen peroxide has been fabricated using the *in situ* oxidative polymerization. The fabricated electrode sensor was successfully used to detect H_2_O_2_ in real samples.

## Graphical abstract

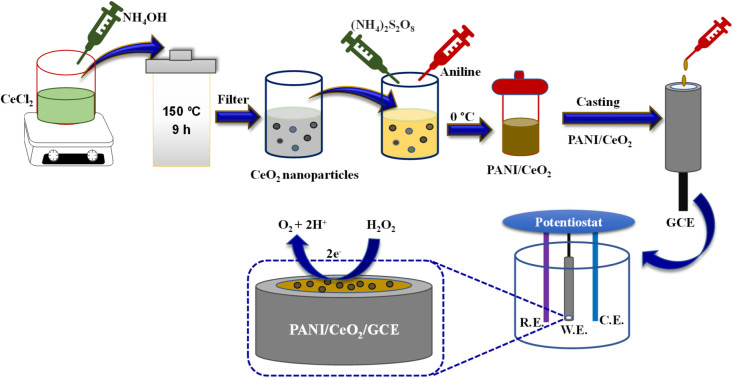

## 1. Introduction

Metal oxides derived from polymer nanocomposites are novel nanostructured materials that have shown customized properties that are seldom observed in pure polymers. Those nanocomposites have demonstrated exceptional performance in a variety of domains, making them appropriate for a broad range of real-world applications.^1–4^ The above multifunctional materials have a wide range of applications, along with the construction of different kinds of sensors.^5–7^ The nanofillers, which seem to be nanoscale in size and have a highly porous structure, are the primary reasons for the vast uses of nanocomposites. The porous structure is responsible for increasing the polymer nanocomposites' physical and chemical characteristics. Because of their good qualities with synergetic or complimentary tendencies among conductive polymers and inorganic nanoparticles, composite materials with conductive characteristics have prospective uses in a variety of disciplines.^8^ There have been many publications on the creation of nanoparticles in polymers. *In situ* polymerization, anodic oxidation, electrochemical, and electrodeposition are some processes employed to create these composite materials.^9–11^ Polyaniline (PANI) has been intensively explored among conjugated polymers over the past two decades due to its unusual electrochemical and physicochemical properties. PANI is likely to be much more explored since it has a huge variety of superior properties deduced from its structural flexibility, as well as some economic benefits such as good environmental stability, processability in aqueous solutions and organic solvents, achievement consistency, distinct optical, electrical, and electrochemical characteristics, and its simple non-redox doping/de-doping ability in acid/base reactions.^12,13^ Along with its chemical and environmental stability, unusually quick redox rate, and acid-base doping/de-doping chemistry, PANI has been regarded among the most promising and effective components. A significant amount of work has gone into the fabrication of PANI electrodes for supercapacitors using different methods such as self-doping, template polymerization, *in situ* polymerization, and counter-ion induced technique.^14–17^ Cerium oxide (CeO_2_) is a promising material, and CeO_2_ nanostructures have a diverse range of potential applications in many areas, including catalysis, fuel cells, optical additives, high-temperature oxidation resistance, cosmetic components, free-radical scavengers, and others.^18–20^ Moreover, it plays a crucial part in the growth of technology for environmental applications.^21–23^

CeO_2_ nanostructures have also been used in sensor technologies. Due to their high specific surface area, good electrochemical activity, and the probability of promoting electron transfer reactions at a lower overpotential, CeO_2_ nanostructures have piqued interest among researchers due to their unique and diverse applications in various areas in many disciplines, particularly sensor advanced technologies.^18,19,24,25^ CeO_2_ nanostructures were created as nanoparticles with small dimensions due to their interesting uses in electronics, energy storage, catalysis, and gas sensing. As a result, in this study, we attempted to create PANI/CeO_2_ nanocomposites by inserting crystalline low-dimensional CeO_2_ nanoparticles into a PANI matrix. The glassy carbon electrode (GCE) modified with the PANI/CeO_2_ nanocomposite was used to detect hydrogen peroxide (H_2_O_2_) in actual biological samples like tap water and packed bottled milk. Field-emission scanning electron microscopy (FE-SEM) was used to analyze the surface morphology of PANI/CeO_2_ nanocomposites, while Fourier transform infrared (FTIR), ultraviolet-visible (UV-vis), and X-ray diffraction (XRD) spectroscopy techniques were used to evaluate the stoichiometry and crystalline structure. Linear sweep voltammetry (LSV), cyclic voltammetry (CV), and electrochemical impedance spectroscopy (EIS) were used to study the electrode detection limit, sensitivity, and pH influence on the detection of H_2_O_2_ using PANI/CeO_2_ nanocomposites.

## 2. Experimental

### 2.1 Materials & solvents

Aniline (BDH, 99%) was freshly distilled before use *via* a simple distillation method. Phosphoric acid (Merck, 97%), and ammonium persulfate (BDH, 99%) was used without further purification. All other used chemicals, such as cerium chloride, ammonium nitrate, *etc.*, were purchased from Aldrich Chemical Co. All the chemicals were of reagent grade and used without further purification. Distilled water was used throughout the study.

### 2.2 Growth of CeO_2_ nanoparticles

CeO_2_ nanoparticles were synthesized by the hydrothermal method in which CeCl_2_ (1.36 g) was dissolved in distilled water (100 ml) with constant stirring for about 30 min at room temperature and were then titrated with NH_4_OH solution until pH = 10.2. The resultant solutions were then transferred into a Teflon autoclave and heated at 150.0 °C for 9 hours. Finally, a white precipitate was obtained, which was washed with water and ethanol several times and dried at room temperature. The resulting white powder was calcined at 250 °C for 5 hours.

### 2.3 Oxidative polymerization of pure PANI

Pure PANI was prepared using a well-known oxidative polymerization technique as previously described in the literature.^26,27^

### 2.4 Fabrication of PANI/CeO_2(1,3,5,10)_ nanocomposites

**Fig. 1:**
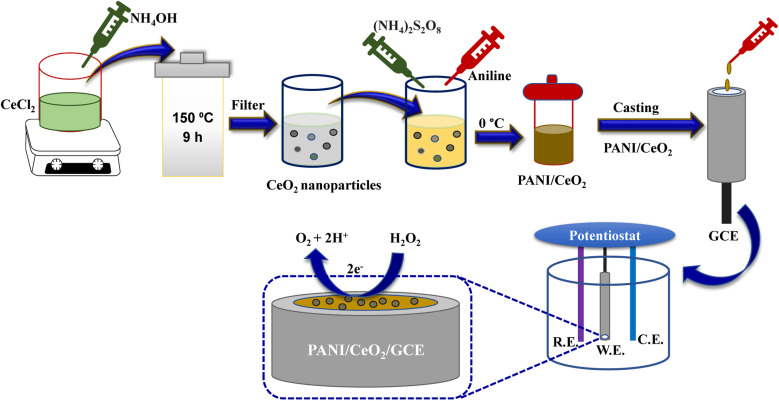
Schematic illustration of PANI/CeO_2_ synthesis and the modification of GCE with the prepared nanocomposite.

The PANI/CeO_2_ nanocomposites were synthesized using a constant weight of aniline and 1, 3, 5, 10 wt% of CeO_2_ nanoparticles. New PANI/CeO_2_ nanocomposites with different CeO_2_ ratios (wt%) were prepared by *in situ* polymerizations in an aqueous solution. In a typical procedure, a certain amount of CeO_2_ nanoparticles (0.01, 0.03, 0.05, 0.1 g) and 1 ml aniline monomer were added to 35 ml of 0.5 mol per L H_3_PO_4_ solution and sonicated for 30 min. Then, 2.78 g ammonium persulfate was dissolved in 20 ml of 0.5 mol per L H_3_PO_4_ solution and added dropwise to the mixture with constant stirring. The polymerization was carried out at 0 °C under nitrogen for 24 h. The composites obtained by filtering were washed respectively with deionized water and ethanol and dried under vacuum at 60 °C for 24 h (Fig. 1). The fabricated nanocomposites were given the following abbreviations PANI/CeO_2(1)_, PANI/CeO_2(3)_, PANI/CeO_2(5)_, PANI/CeO_2(10)_ for the different CeO_2_ loadings (1, 3, 5, 10 wt%) within the polymer matrix.

### 2.5 PANI/CeO_2(10)_ nanocomposite-modified electrode fabrication

Glassy carbon electrodes were polished using a velvet pad with 0.05 μm alumina slurry, followed by sonication in acetone and washing with double distilled water (DDW) at room temperature (25 ± 3 °C) (RT). A fine dispersion of 1%, 3%, 5%, and 10% of CeO_2_ loading in the form of PANI/CeO_2(1,3,5,10)_ was prepared by dissolving 20 mg of each in 1 ml DDW by sonicating the solution mixture for up to 3 h. Next, 20 μL of each nanocomposite dispersion was deposited on a GCE, which was assigned as a non-enzymatic electrochemical sensor electrode for the detection of H_2_O_2_ (Fig. 1). Electrochemical studies of the modified GCE deposited with different concentrations of PANI/CeO_2(1,3,5,10)_ were conducted at room temperature using an Autolab potentiostat (Metrohm PGSTAT302N-AUT85887) fitted with an impedance analyzer (FRA32M.X) and programmed with Nova 1.1 software. A conventional cell having a three-electrode system, including a working electrode (bare or GCE, 3 mm diameter), reference electrode (Ag/AgCl), and counter electrode (platinum wire), was used to perform a CV, LSV, and EIS in the potential range of −0.6 to 1.0 V at the scan rates of 20 to 200 mV s^−1^. The optimization procedures were carried out on PANI/CeO_2(10),_ which gave the highest electrochemical performance among all the fabricated materials.

### 2.6 Instrumentation

The synthesized nanoparticles and nanocomposites were characterized in detail in terms of their structural, optical, and sensing properties. XRD diffractograms were taken with a computer-controlled X'Pert Explorer, PANalytical diffractometer. The surface morphology of the nanohybrid membranes was studied at 15 kV using a JEOL scanning electron microscope (JSM-7600F, Japan). FT-IR spectra were recorded in KBr dispersion in the range of 400 to 4000 cm^−1^ on a PerkinElmer (spectrum 100) FT-IR spectrometer. UV-vis spectra were recorded from 250–700 nm using a PerkinElmer (Lambda 950) UV-visible spectrometer. The electrochemical measurements were carried out by using an Autolab potentiostat (Metrohm PGSTAT302N-AUT85887) fitted with an impedance analyzer (FRA32M.X) and programmed with Nova 1.1 software.

## 3. Results and discussion

### 3.1 Physicochemical characterization of CeO_2_ nanoparticle and PANI/CeO_2(1,3,5,10)_ nanocomposites

**Fig. 2:**
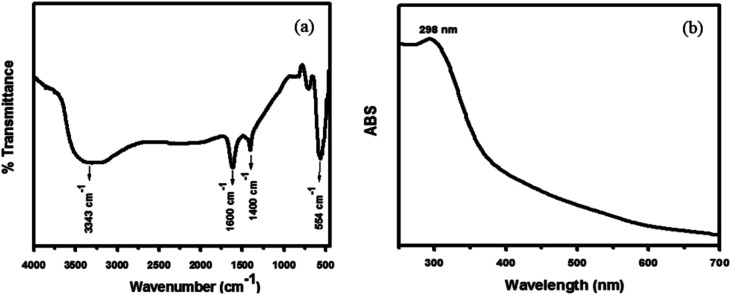
Typical FT-IR spectrum of the synthesized CeO_2_ nanoparticles (a) and typical UV-vis spectrum of the synthesized CeO_2_ nanoparticles (b) grown *via* a simple hydrothermal process.

Hybrid PANI/CeO_2_ nanocomposites have been fabricated using the familiar *in situ* oxidative polymerization process and applied in the electrochemical sensing of hydrogen peroxide. PANI/CeO_2(1,3,5,10)_ nanocomposites-based modified glassy carbon electrodes were utilized as electrochemical sensors for the detection of hydrogen peroxide. Prior to the fabrication process, CeO_2_ nanoparticles were prepared by a hydrothermal method and the chemical structure was confirmed by common characterization tools including FT-IR, UV-vis, XRD and FE-SEM measurements. The formation of CeO_2_ nanoparticles was confirmed by the FT-IR spectrum, which is shown in Fig. 2a. FT-IR showed an absorption band at 554 cm^−1^, which is the characteristic peak for the Ce–O stretching vibration. FT-IR also showed absorption bands at 3343 cm^−1^, 1600 cm^−1^and 1400 cm^−1^, responsible for water and CO_2_, which nanocrystalline materials normally absorb from the environment due to their high surface-to-volume ratio.^25^

The UV-vis spectra of the synthesized nanoparticles were also examined to check the photocatalytic activity of CeO_2_ nanoparticles, which depends on their optical properties. As shown in Fig. 2b, the UV-vis spectra of the CeO_2_ nanoparticles exhibited a well-defined absorption band at 298 nm.^25^ Usually, a peak at 298 nm corresponds to the fluorite cubic structure of CeO_2_. The UV spectrum showed no other peak related to impurities and structural defects, confirming that the synthesized nanoparticles were pure CeO_2_.

**Fig. 3:**
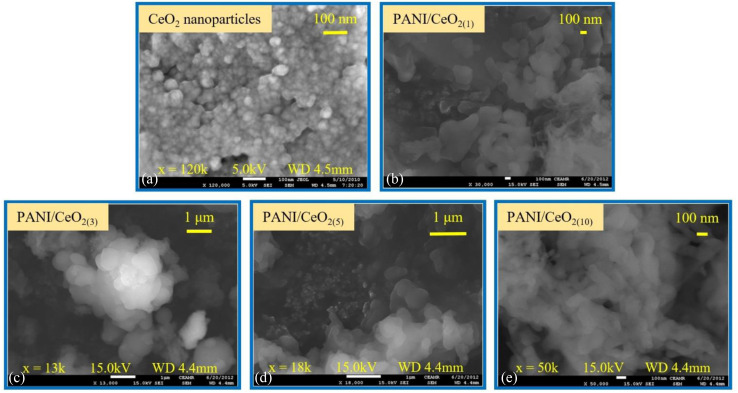
Typical FESEM images of the synthesized CeO_2_ nanoparticles grown *via* a simple hydrothermal process (a) and for the fabricated PANI/CeO_2(1,3,5,10)_ nanocomposites (b–e).

FE-SEM was used to characterize the structure and shape of the CeO_2_ nanoparticles as demonstrated in Fig. 3a. The FE-SEM images show that the synthesized product consists of aggregated spherical particles, grown with high density. The average diameter of the grown nanoparticles was about 50 ± 10 nm. The surface images of the PANI/CeO_2(1,3,5,10)_ nanocomposites indicate that the CeO_2_ nanoparticles were homogeneously distributed in the PANI without any aggregation (Fig. 3b–e), and the data is in good agreement with the previous reports in the literature.^7^

The main target of this study is to fabricate a set of PANI/CeO_2_ nanocomposites in the form of PANI/CeO_2(1,3,5,10)_ containing different loadings of the pre-prepared CeO_2_ nanoparticles (weight%) inside the polymer host matrix. Here, 1, 3, 5, and 10% of the CeO_2_ nanoparticles have been inserted into the pure PANI to form the required PANI/CeO_2_ nanocomposites.

**Fig. 4:**
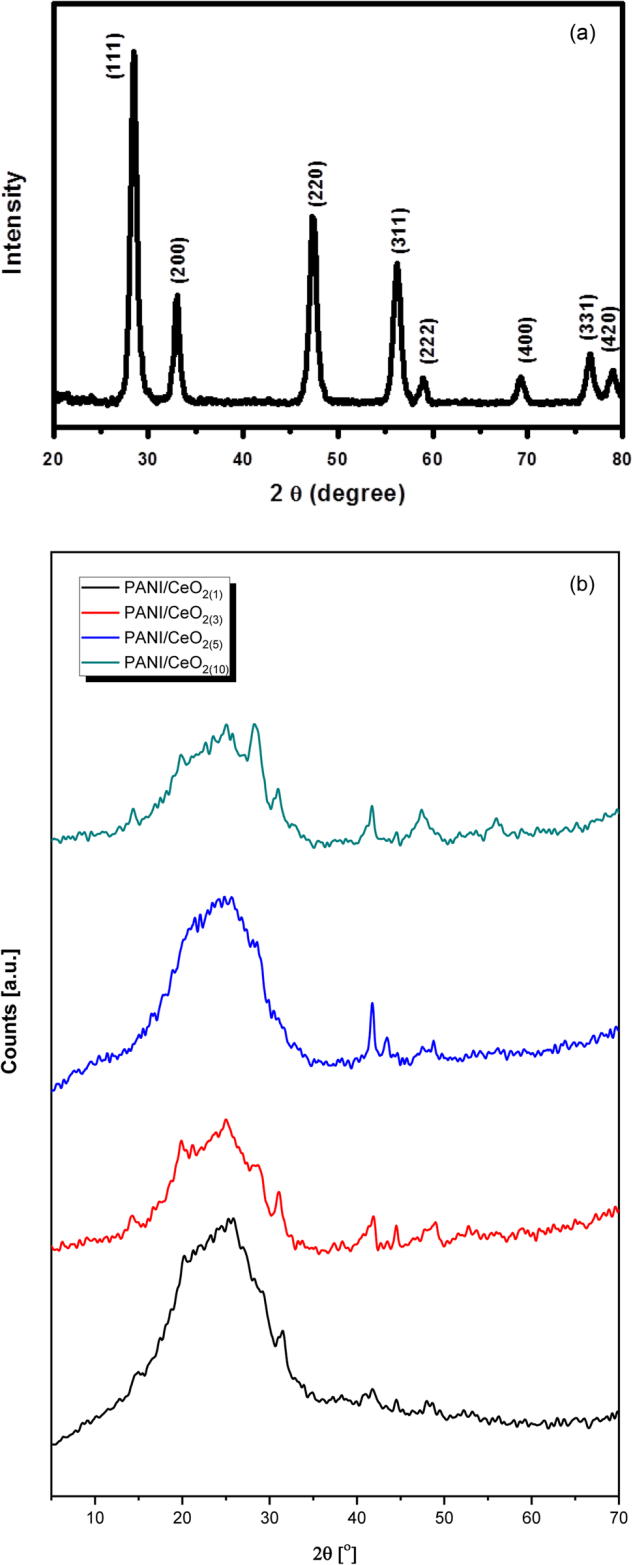
(a)Typical XRD pattern of the synthesized CeO_2_ nanoparticles grown *via* a simple hydrothermal process. (b) Typical XRD patterns of the fabricated PANI/CeO_2(1,3,5,10)_ nanocomposites.

Fig. 4a represents the wide-angle XRD pattern of CeO_2_ nanoparticles. The XRD pattern exhibited well-defined peaks located at 2*θ* = 28.5, 33.1, 47.5, 56.2, 59.0, 69.4, 76.6 and 79.0, corresponding to (111), (200), (220), (311), (222), (400), (331) and (420) planes, respectively. All these peaks of the synthesized CeO_2_ can be indexed to the cubic phase of CeO_2_ (JC-PDF cards no 01-075-8371) because all the peaks are well-matched with the standard peaks of crystalline cubic CeO_2_.^25^ According to the JC-PDF cards, the lattice parameter a is 5.4116 Å for CeO_2_ nanoparticles. All the obtained peaks in the pattern belong only to CeO_2_. No other peak related to impurities was detected within the XRD's detection limit, which confirmed that the obtained nanoparticles are pure CeO_2_ with a cubic phase.

The XRD pattern of the pure PANI and the fabricated PANI/CeO_2(1,3,5,10)_ nanocomposites (Fig. 4b) shows a peak with a peak maximum at 25.0 Å, which is responsible for the amorphous phase of PANI. The amorphous nature of pure PANI is in agreement with previously reported studies.^26,27^ The XRD patterns of the fabricated materials also showed several small peaks attributed to the CeO_2_ in the nanocomposites.^7^ The CeO_2_^–^related peak intensities were noticeably increased, in agreement with the increased CeO_2_ loading in the formation of the nanocomposite. All diffraction peaks in the obtained patterns confirmed that the synthesized products are nanocomposites of PANI and CeO_2_ nanoparticles. XRD did not show any other peaks except PANI and CeO_2_, verifying that the synthesized nanocomposites are made of PANI and CeO_2_.^7,26,27^

**Fig. 5:**
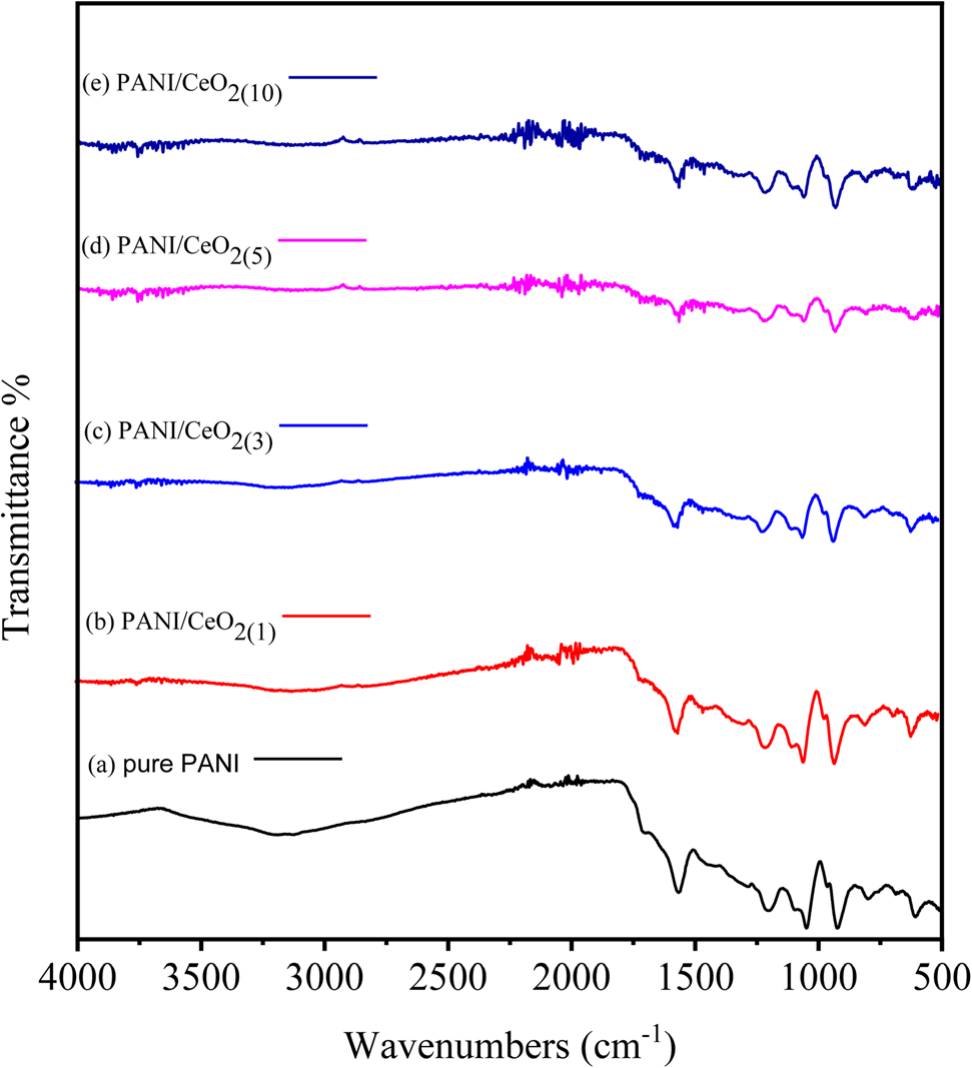
FTIR spectra of pure PANI (a) and PANI/CeO_2(1,3,5,10)_ nanocomposites (b–e) with different loadings.

The pure PANI, and the fabricated PANI/CeO_2(1,3,5,10)_ nanocomposites were further characterized by FTIR spectroscopy, as illustrated in Fig. 5. Samples were examined over a wide range from 4000 to 500 cm^−1^. The peaks of pure PANI observed, including broad peaks in the range of 3037–3319 cm^−1^, were due to N–H bond stretching vibrations of secondary amino groups,^28,29^ and other peaks observed at 1568 cm^−1^ and 1043 cm^−1^ were attributed to the C=C stretching of the quinoid ring in PANI, and (N=Q=N).^30,31^ Moreover, benzene rings were identified by C–N and C–C peaks at 1304 and 1391 cm^−1,^ respectively.^32,33^ The absorption band observed at 1615 cm^−1^ is due to the “scissor’’ bending of related water.^34^ For the PANI/CeO_2(1,3,5,10)_ nanocomposites, new peaks were observed at 822, 754, and 583 cm^−1^ in the FTIR spectra of each composite, attributed to the Ce–O asymmetric stretching mode and bending vibrations. The Ce–N symmetric stretching vibration of Ce–N caused the peak at 502 cm^−1,^ which shows that the composite was successfully formed with PANI.^33,34^ The concentration percentage varied, as can be seen. This refers to each bond, although no shift in the location of the bonds was observed. The FT-IR spectrum after adding PANI to CeO_2_ in the form of PANI/CeO_2(1,3,5,10)_ nanocomposites is displayed in Fig. 5a–e. Some IR peaks of PANI have shifted to lower wavenumber with reduced intensity, which may be due to the formation of hydrogen bonds between the hydroxyl groups on the surface of the CeO_2_ nanoparticles and the imine groups in the polyaniline molecular chains.^35^ A similar type of FTIR behavior with reduced intensity of IR peaks was also reported by Sasikumar *et al.* (2015) for the PANI/CeO_2_ nanocomposite caused due to the hydrogen bonding between hydroxyl groups on the surface of the CeO_2_ nanoparticles and the polyaniline's imine groups.^36^

### 3.2 Electrochemical performance of PANI/CeO_2(1,3,5,10)_ nanocomposites

**Fig. 6:**
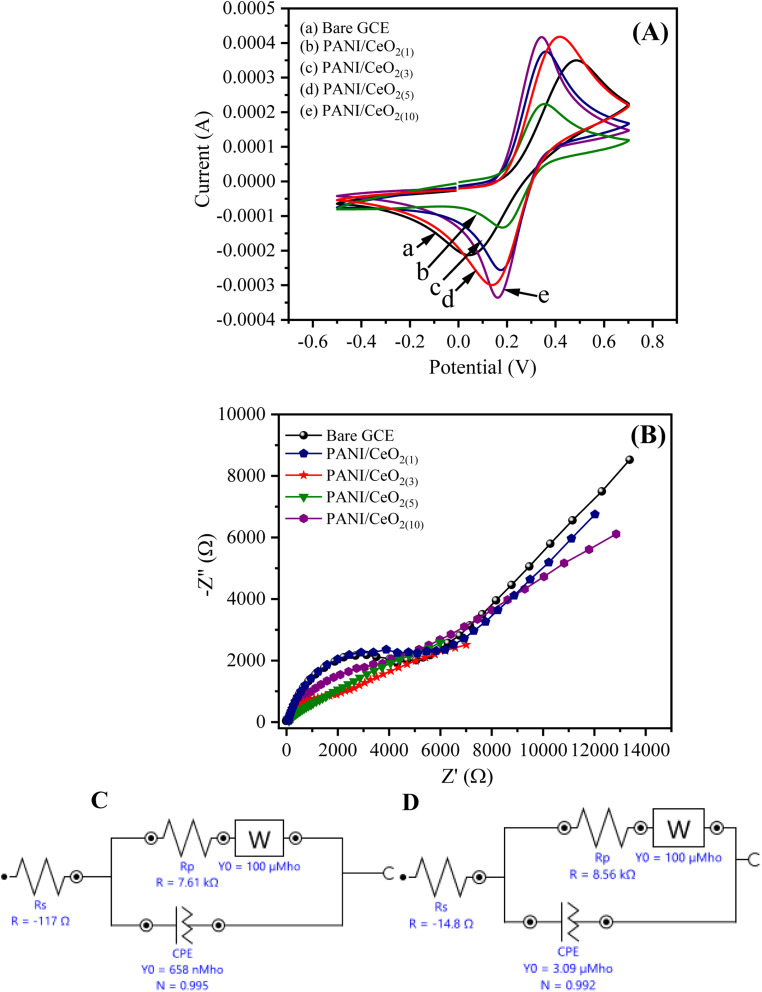
(A) The obtained cyclic voltammogram and (B) EIS curves of the bare GCE, and PANI/CeO_2(1,3,5,10)_-modified GCE response in 0.1 mM K_3_Fe(CN)_6_ and equivalent circuit models (C) for PANI/CeO_2(1)_ (D) PANI/CeO_2(10)_ nanocomposite GCE electrodes.

The cyclic voltammograms (CV) of the bare GCE, PANI/CeO_2(1,3,5,10)_ nanocomposites-modified GCE in 0.1 M K_3_Fe(CN)_6_ (prepared in 0.1 M KCl) at the applied potential of −0.5 to 0.7 at the scan rate of 100 mV s^−1^ are shown in Fig. 6. The CV curves indicate that PANI/CeO_2(1,3,5,10)_ nanocomposites-modified GCE with concentrations of 1% to 10% have larger electro-active area and high current density as compared to bare GCE. The surface area of the electrode has a significant role in the rate of reaction and it was found to be larger in the case of PANI/CeO_2(1,5,10)_ nanocomposites-modified GCE than bare GCE, which produced a high CV current. Therefore, the PANI/CeO_2(1,3,5,10)_ nanocomposites promote electron transfer using potassium ferricyanide as a supporting electrolyte. It was also observed that with increasing the concentration of cerium oxide, the value of the anodic peak current also increased (Fig. 6A). The CV curve of the PANI/CeO_2(10)_ nanocomposites-modified GCE indicated that the further increase in the value of the anodic peak current may be due to feasible electron transfer among counter and working electrodes.

EIS is considered a significant study for demonstrating the resistivity or conductance between the electrolyte interface and GCE. The value of the negative imaginary impedance can report this (−Z′′) *vs.* real impedance (Z′) in Nyquist plots. Fig. 6B shows the Nyquist plot for PANI/CeO_2(1,3,5,10)_ nanocomposites-modified GCE. The EIS equivalent circuit models used for determining the values of charge transfer resistance (*R*_ct_), solution resistance (*R*_s_), constant phase element (CPE), and Warburg impedance (W) for PANI/CeO_2(1),_ and PANI/CeO_2(10)_-modified GCE are shown in Fig. 6C and D. The Nyquist plot of PANI/CeO_2(10)_-modified GCE showed a reduced diameter of the semi-circle in the lower frequency region. The shape of the semi-circular obtained in the higher frequency region indicated a limited electron transfer process for the reaction process while the diffusion process is allied with a diffusion-reaction process. The reduced shape of the semi-circle in the lower frequency region demonstrates a faster rate of electron transfer, therefore, less resistivity with improved conductivity.^37^Fig. 6(C and D) show that *R*_ct_ (*R*_p_) values for the PANI/CeO_2(10)_ and PANI/CeO_2(1)_-modified GCE were 7.61 and 8.56 kΩ, respectively. The lower value of *R*_ct_ also confirmed the improved electron transfer rate for the PANI/CeO_2(10)_-modified electrode. The obtained results of both CV and EIS studies suggested the high anodic peak current and improved rate of electron transfer for the PANI/CeO_2(10)_-modified electrode in comparison to the other concentrations; therefore, further electrochemical characterizations were carried out with only a modified electrode having the PANI/CeO_2(10)_ nanocomposite.

#### 3.2.1 CV response under different pH values

**Fig. 7:**
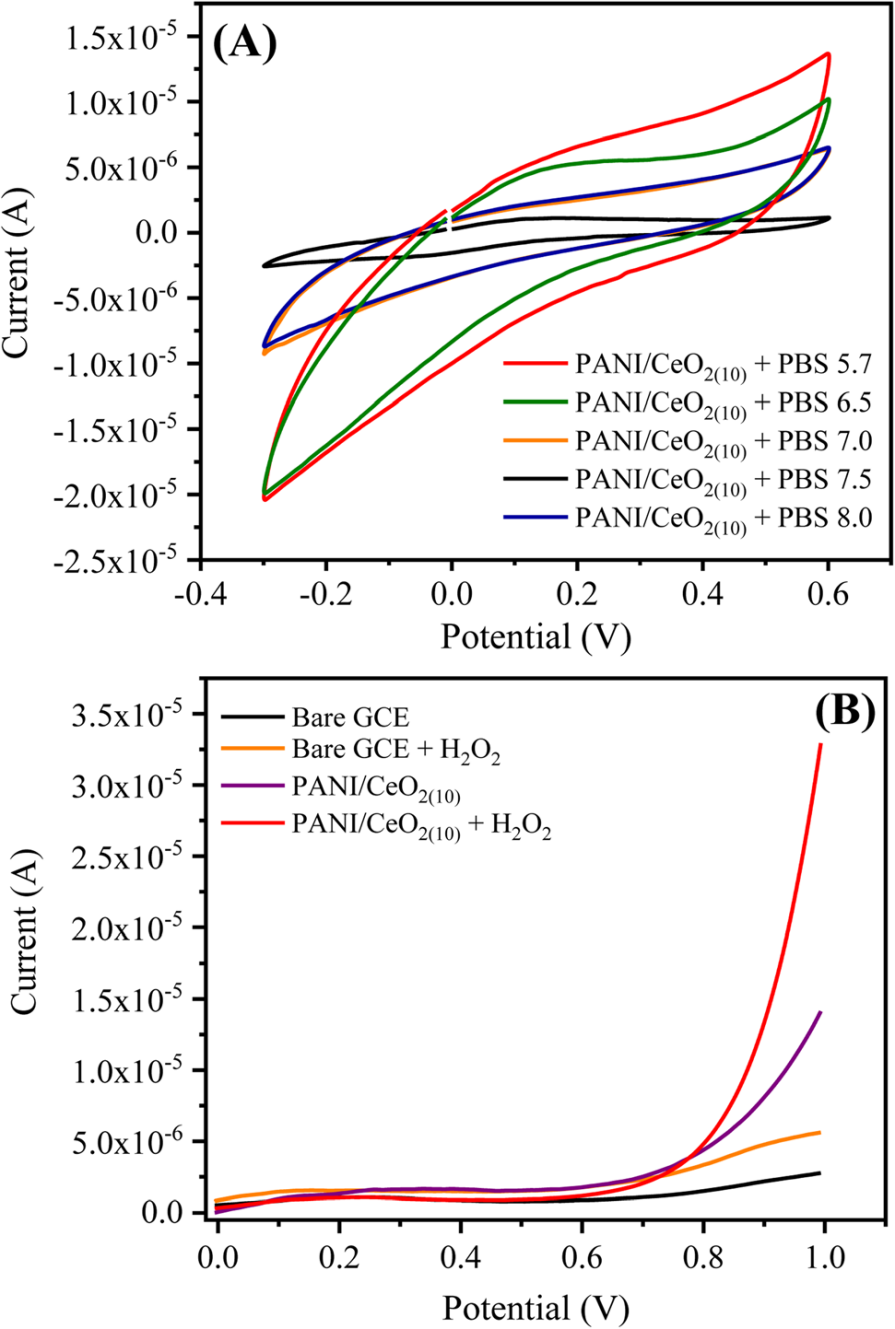
(A) CV response of the PANI/CeO_2(10)_ nanocomposite GCE using phosphate buffer solutions in different pH ranges, and (B) the current responses of the bare and PANI/CeO_2(10)_ nanocomposite GCE in the absence and presence of 0.1 mM H_2_O_2_ using PBS 5.7 at 100 mV s^−1^.

To investigate the optimized supporting electrolyte for the efficient oxidation of hydrogen peroxide on the surface of the PANI/CeO_2(10)_-modified GCE, the CV response was recorded using 0.1 M phosphate buffer solutions (PBS) with different pH values such as 5.7, 6.5, 7.0, 7.5 and 8.0 (Fig. 7A). The obtained CV response showed that the highest anodic current density of PANI/CeO_2(10)_-modified GCE was observed in PBS 5.7, while the lowest anodic current density was with PBS 7.5. This reveals that the PANI/CeO_2(10)_-modified GCE enhanced the catalytic oxidation of H_2_O_2_ in an acidic buffer solution. Therefore, the PANI/CeO_2(10)_-modified GCE was considered stable under PBS 5.7 and utilized for the sensing of hydrogen peroxide and its useful studies. The electrochemical behaviors of bare and PANI/CeO_2(10)_-modified GCE towards 0.1 mM H_2_O_2_ are shown in Fig. 7B. The linear sweep voltammetry (LSV) curve shows that the PANI/CeO_2(10)_-modified GCE provided high current density while the bare GCE gave very low current under the same applied voltage. The nanocomposite-modified electrode had a sharp and improved rise in the oxidation current that may be due to the enhanced mobility of electrons on the PANI/CeO_2(10)_ electrode, which finally catalyzed the electro-oxidation of H_2_O_2_.^38^ With the addition of H_2_O_2_, the oxidation current enhancement was about 400% as compared to bare GCE; however, it might be inferred that the oxidation of H_2_O_2_ was achieved at lower energy rather than the bare electrode. This analysis also suggested that the oxidation of H_2_O_2_ on the PANI/CeO_2(10)_-modified electrode started at a lower voltage than the bare electrode.

#### 3.2.2 The effect of scan rate

**Fig. 8:**
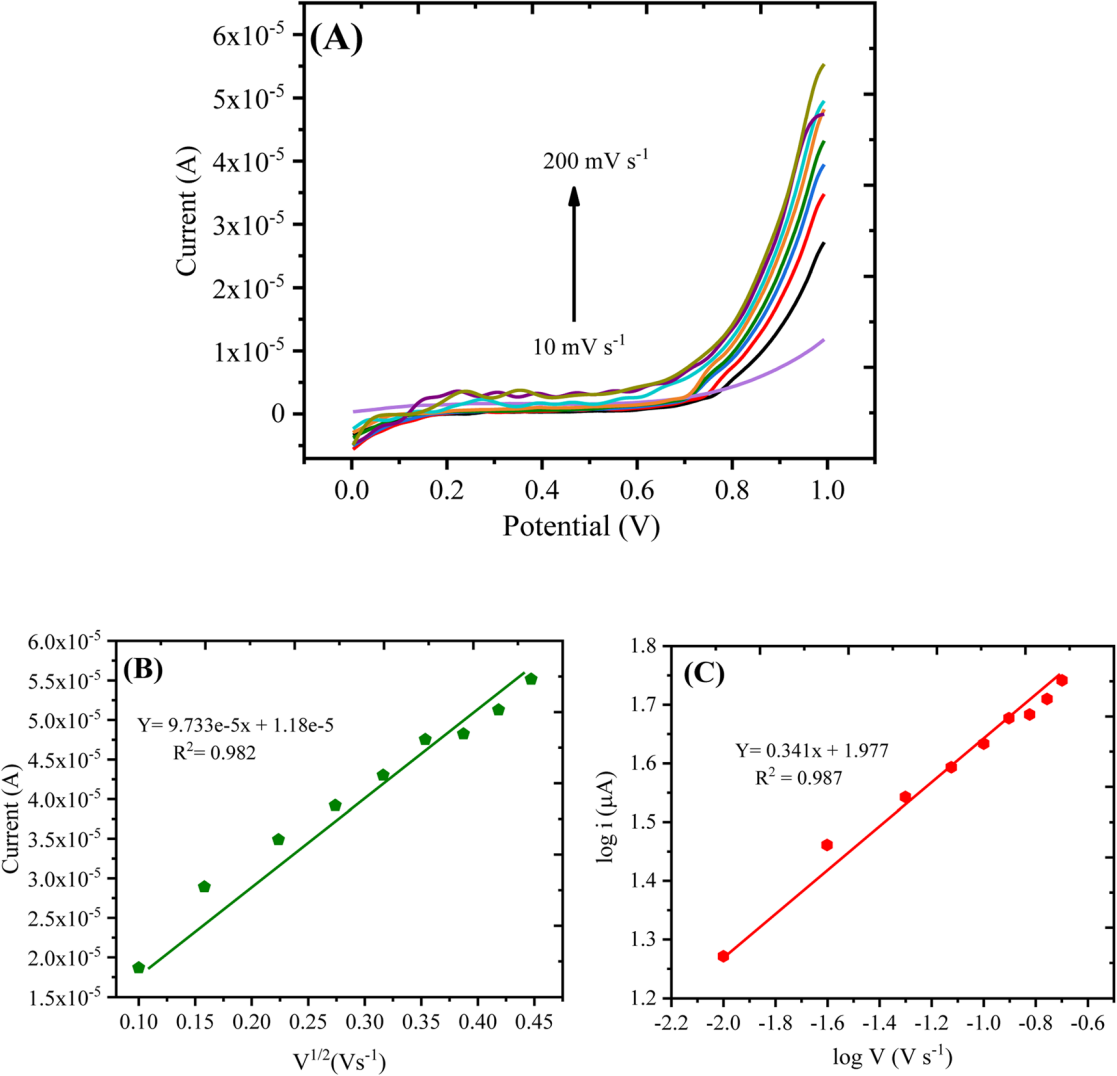
(A) LSV curve showing the scan rate effect on the oxidation current of 0.1 mM H_2_O_2_. (B) Oxidation current plotted against v^1/2^ and (C) the logarithm of the oxidation current plotted against the logarithm of the scan rate.

The influence of the scan rate on the LSV curve with varying the scan rate of the PANI/CeO_2(10)_ nanocomposite-modified GCE in PBS 5.7 and 0.1 mM hydrogen peroxide is shown in Fig. 8A. It was found that on increasing the scan rate from 10 to 200 mVs^−1^, the intensity of the oxidation current increased linearly. Fig. 8B shows a linear graph, which was obtained by plotting the oxidation current values against the square roots of different scan rates (*V*^1/2^) as articulated by eqn (1) (Fig. 8B);

1 *i*_max_ = 0.0000977 × *V*^1/2^ + 0.0000118 (*R*^2^ = 0.982)

The linear graph with varying scan rates suggested that the current was proportional to *V*^1/2^, which shows that the redox process was diffusion-controlled. Moreover, to demonstrate whether the oxidation reaction was adsorption or diffusion-controlled, another plot was obtained through the logarithm of anodic current *vs.* logarithm of the scan rate as shown in Fig. 8C, expressed by the following eqn (2):

2 log *i*_max_ = 0.341 × log *v* + 1.977 (*R*_2_ = 0.987)

As per the previous reports,^39,40^ the slope of 0.5 is related to the diffusion-controlled process, and that centered around 1.0 denotes an adsorption-controlled reaction. For the current work, the obtained slope value (0.341) was close to 0.5; therefore, the oxidation of hydrogen peroxide on the surface of PANI/CeO_2(10)_ nanocomposite-modified GCE can be assigned as a diffusion-controlled reaction.

#### 3.2.3 The performance and reaction mechanisms of the PANI/CeO_2(10)_ nanocomposite-modified GCE sensor

**Fig. 9:**
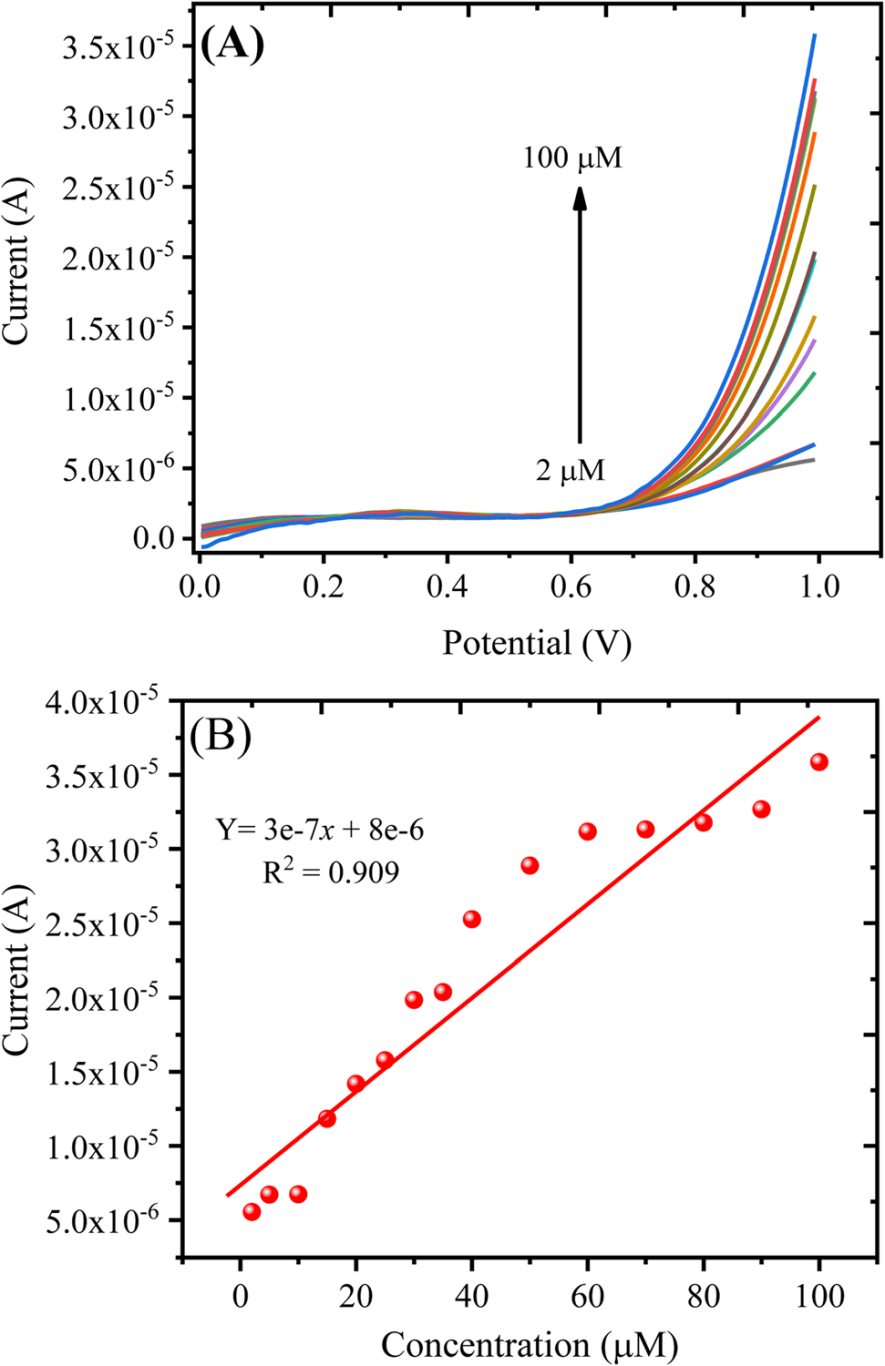
(A) LSV response obtained with the PANI/CeO_2(10)_ nanocomposite-modified GCE in varying concentrations of H_2_O_2_. (B) A plot of oxidation current against the concentration of H_2_O_2_.

The LSV technique was chosen for the assay of H_2_O_2_ due to its high accuracy and sensitivity toward an irreversible reaction process.^41,42^ In LSV, the potential is linearly varied, and the respective current value at every potential is recorded. Therefore, the PANI/CeO_2(10)_ nanocomposite-modified GCE was used to demonstrate the concentrations of hydrogen peroxide under optimal conditions through the LSV technique, and it was observed that with the increase in the concentration of H_2_O_2,_ the intensity of current for the modified electrode increased. Fig. 9A shows the LSV curve, where it can be observed that before the addition of hydrogen peroxide, the LSV response was very low (1.0 v), but after the addition of 2 μM, the small increase in the oxidation current was found, which continued to increase as the concentration of hydrogen peroxide increased. The peak current *vs.* concentration or equation of the plot (Fig. 9B) is given as eqn (3):

3 *i*_max_ = 3e − 7 × *c*(μM) + 8e − 6 (*R*_2_ = 0.909)

The detection of limit (LOD) can be measured using the formula: LOD = 3*S*/*b*, where *S* represents the standard deviation of the intercept, and *b* represents the calibration plot slope. The estimated LOD value for the PANI/CeO_2(10)_ nanocomposite-modified GCE towards H_2_O_2_ was 0.1484 μM. The quantification limit (LOQ) was measured using the formula: LOQ = 10*S*/*b*. The measured value for LOQ was 0.49 μM. The calibration plot was plotted up to the addition of 100.0 μM, which suggested the linear dynamic range for the proposed sensor was from 2 to 100.0 μM.

By applying the Randles-Sevcik equation,^43^ the EASA of the PANI/CeO_2(10)_ nanocomposite-modified GCE was calculated by recording the CV (Fig. 6A(e)) in the [Fe(CN)_6_]^3−^/^4−^ redox couple.

4 *I*_p_ = 2.69 × 10^5^ × *A* × *n*^3/2^ × *D*^1/2^ × *C* × *γ*^1/2^

*I*_p_ is the peak current (Ampere), *A* refers to the electrochemically active surface area (cm^2^), and *D* is the diffusion coefficient of 0.5 mM [Fe(CN)_6_]^3−/4−^ (6.70 × 10^−6^ cm^2^ s^−1^), *C* is the concentration of the [Fe(CN)_6_]^3−/4−^ (mol L^−1^), *n* is the number of transferred electrons for the [Fe(CN)_6_]^3−/4−^ redox couple (*n* = 1), and *γ* is the scan rate (V s^−1^). Accordingly, the EASA of the PANI/CeO_2(10)_ nanocomposite-modified GCE was calculated to be 0.0387 cm^2^. The surface area of the commercially obtained bare GCE electrode was 0.0316 cm^2^ (as given by the manufacturer); upon the deposition of the PANI/CeO_2(10)_ nanocomposite, the EASA of the modified GCE was enhanced. This increment in EASA is believed to have aided the modified GCE's electrocatalytic and conductive properties.

The electrocatalytic mechanism for detecting H_2_O_2_ using the PANI/CeO_2(10)_ nanocomposite-modified GCE is based on the electrochemical oxidation of H_2_O_2_ to O_2_ under applied potential. As per previous reports, in CeO_2_ nanoparticles, the anti-oxidation behavior of the active centers can be demonstrated by surface Ce sites that can coordinate with the O_2_ sites.^44,45^ For the PANI/CeO_2(10)_ nanocomposite-modified GCE, the probable active centers are Ce^4+^ and Ce^3+^ and O_2_ vacancies. As the H_2_O_2_ is incorporated into the working solution, ceria behaves like a nanozyme; the adsorption of H_2_O_2_ stimulates the electrochemical process under applied potential by sharing electrons. It was presumed that the mechanism of oxidation for H_2_O_2_ on the PANI/CeO_2(10)_ nanocomposite-modified GCE can be represented by reaction 5.^46^

5 H_2_O_2_ O_2_ + 2e^−^ + 2H^+^

#### 3.2.4 Stability of PANI/CeO_2(10)_ nanocomposite-modified GCE

**Fig. 10:**
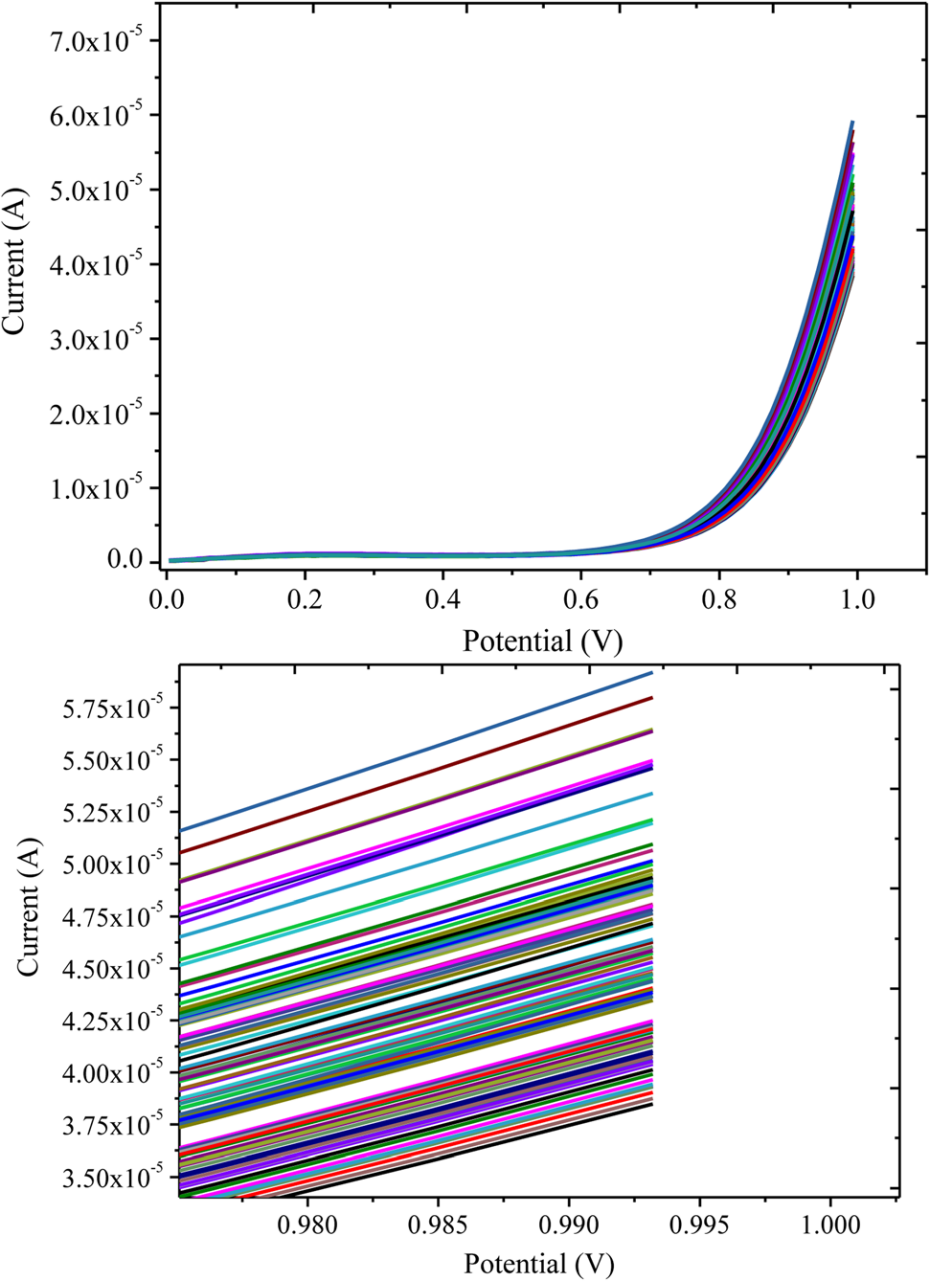
LSV response of the PANI/CeO_2(10)_ nanocomposite-modified GCE in 0.1 mM H_2_O_2_ for multiple measurements.

The stability of the PANI/CeO_2(10)_ nanocomposite-modified GCE towards the detection of H_2_O_2_ was determined in terms of repeating the 100 current response measurements under optimal conditions in 0.1 mM H_2_O_2_ (Fig. 10). The results showed that the developed PANI/CeO_2(10)_ nanocomposite-modified GCE showed repeatable current response with a decrease in the current response of 29.85%, which confirmed that the current response was satisfactorily repeatable for the proposed electrode.

#### 3.2.5 Interferent effects of common molecules and ions

**Fig. 11:**
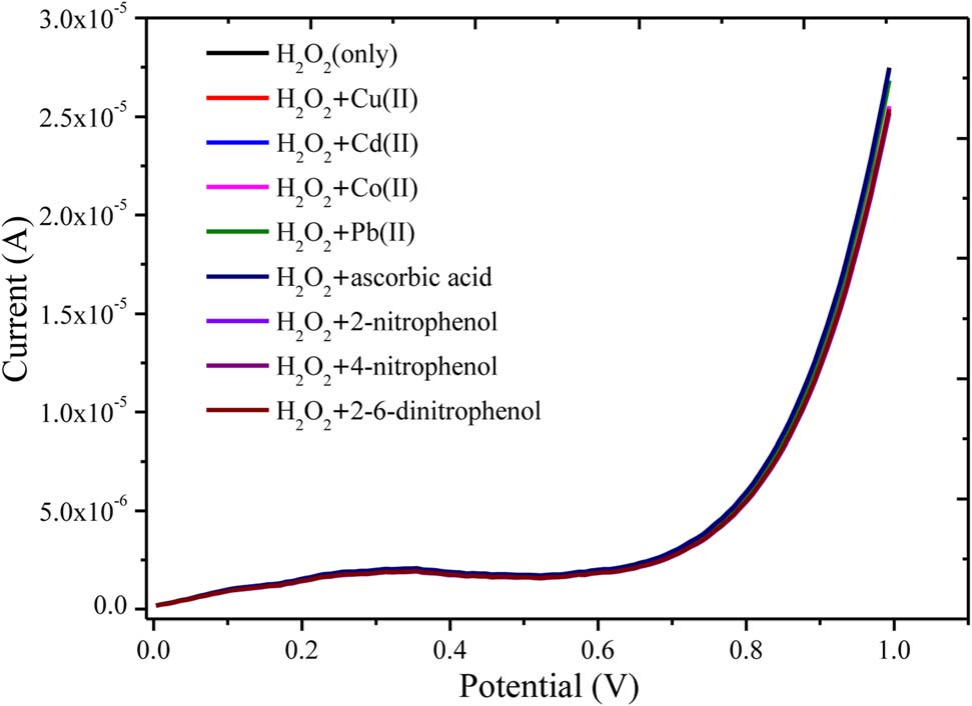
LSV response of the PANI/CeO_2(10)_ nanocomposite-modified GCE in 50 μM of H_2_O_2_ and interferent inorganic ions and organic molecules such as Cu^2+^, Cr^6+^, Cd^2+^, Co^2+^, Pb^2+^, ascorbic acid, 2-nitrophenol, 4-nitrophenol, and 2,4 dinitrophenol.

The effects of interferent inorganic ions and organic molecules on the detection of H_2_O_2_ have been investigated to examine the anti-interference aptitude of the PANI/CeO_2(10)_ nanocomposite-modified GCE. The LSV current responses of the PANI/CeO_2(10)_ nanocomposite-modified GCE in the presence of 50 μM H_2_O_2_ containing a 3-fold concentration of likely interferents such as Cu^2+^, Cr^6+^, Cd^2+^, Co^2+^, Pb^2+^, ascorbic acid, 2-nitrophenol, 4-nitrophenol, and 2,4 dinitrophenol, were observed (Fig. 11). It was noticed that the mixing of interferents had no obvious influences on the current response of the PANI/CeO_2(10)_ nanocomposite-modified GCE. Therefore, the obtained result suggested that the proposed PANI/CeO_2(10)_ nanocomposite-modified GCE could be considered as highly selective towards the determination of hydrogen peroxide.

### 3.3 Real sample analysis

The applicability and suitability of the PANI/CeO_2(10)_ nanocomposite-modified GCE for the detection of H_2_O_2_ in real samples were investigated through the standard addition method (spiking) as reported elsewhere.^47^ For this, 10 μM H_2_O_2_ was spiked into the milk and tap water samples, and the recovery analysis was carried out. For this investigation, 2.5 ml of real samples were diluted with 22.5 ml of 0.1 M PBS 5.7 for matrix matching. Percentage recovery was then measured using eqn (6). The recovery percentage of hydrogen peroxide in real samples (milk and tap water) ranges from 97 to 103, as presented in Table 1.

**Table 1:** Biological sample determination using the PANI/CeO_2(10)_ nanocomposite-modified GCE

Samples	No. of Repeats	Added (μM)	Detected (μM)	Recovery (%)
Milk	1	10	9.80	98
	2	10	10.10	101
	3	10	10.30	103
Tap water	1	10	9.70	97
	2	10	9.90	99
	3	10	9.80	98

The method used was standard addition.6
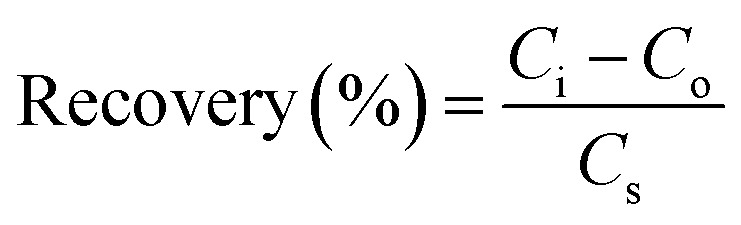


*C*_i_ is the total concentration of analyte, *C*_o_ stands for the concentration of analyte (before spike), and *C*_s_ indicate the concentration of the spiked H_2_O_2_.

Table 2 summarizes the sensing probabilities of the fabricated PANI/CeO_2(10)_ nanocomposite-modified GC as compared to the other investigated electrode systems for detecting hydrogen peroxide.

**Table 2:** A comparison of the PANI/CeO_2(10)_ nanocomposite-modified GCE's sensing performance toward hydrogen peroxide with other sensors reported in the literature

S.No.	Electrode materials	Linear range	Detection limit	Ref.
1	Chitosan/Au nanoparticle	2.0–30.7 μM	0.532 μM	48
2	PANI-CeO_2_	50–500 mM	50 mM	49
3	Methylene blue/SiO_2_	0.01–1.2 mM	4 μM	50
4	ZrO_2_	0.02–9.45 mM	2 μM	51
5	Nb_2_O_5_	0.1–100 μM	0.01 mM	52
6	Pt/carbon nanotube	5–25000 μM	1.5 μM	53
7	Ag-nanoparticle/graphene oxide	100–20000 μM	1.9 μM	54
8	Graphene-AuNPs	20–280 μM	6.0 μM	55
9	Cu_2_O microcubes	5–1500 μM	1.5 μM	56
10	PANI/CeO_2(10)_	0.2–100 μM	0.1484 μM	Present work

## 4. Conclusion

We have reported the fabrication of PANI/CeO_2(1,3,5,10)_, followed by deposition on GCE, for use as an electrochemical sensor for the sensitive detection of H_2_O_2_. CeO_2_ nanoparticles were prepared by a simple hydrothermal method at low temperatures. The detailed morphology of the synthesized nanoparticles was characterized by XRD, FESEM, FTIR, and UV-vis spectra and it was revealed that the synthesized nonmaterial are well-crystalline nanoparticles possessing a cubic structure. The CeO_2_ nanoparticles have been reinforced as a nanofiller to fabricate a set of PANI/CeO_2(1,3,5,10)_ nanocomposites. To confirm the structures, the nanocomposites were structurally characterized by FTIR, XRD, and FESEM measurements. The electrochemical performance of the prepared materials was investigated in detail for hydrogen peroxide detection and determination. The CV results indicated that the increased concentration of CeO_2_ in the PANI polymer matrix from PANI/CeO_2(1)_ to PANI/CeO_2(10)_-modified GCE improved the electron transfer rate and produced a high anodic peak current. The PANI/CeO_2(10)_ nanocomposite-modified GCE demonstrated good performance in terms of relative standard deviation (6.36%) and good limit of detection (0.1484 μM), and showed a linear response in the range from 2 μM to 100 μM. The results confirmed that the proposed PANI/CeO_2(10)_ nanocomposite-modified GCE has good stability and reproducibility with high sensitivity for the detection of H_2_O_2_ in real samples (milk).

## Conflicts of interest

There are no conflicts to declare.

## Supplementary Material

## References

[cit1] Seo J., Jeon G., Jang Eu. S., Bahadar Khan S., Han H. (2011). J. Appl. Polym. Sci..

[cit2] Khan S. B., Alamry K. A., Marwani H. M., Asiri A. M., Rahman M. M. (2013). Composites, Part B.

[cit3] Alamry K. A., Khan A., Hussein M. A. (2022). Synth. Met..

[cit4] Tian B., Zhao L., Li R., Zhai T., Zhang N., Duan Z., Tan L. (2020). Anal. Chem..

[cit5] Wen T., Xia C., Yu Q., Yu Y., Li S., Zhou C., Sun K., Yue S. (2022). Analyst.

[cit6] Rasheed T., Rizwan K. (2022). Biosens. Bioelectron..

[cit7] Khan S. B., Alamry K. A., Bifari E. N., Asiri A. M., Yasir M., Gzara L., Ahmad R. Z. (2015). J. Ind. Eng. Chem..

[cit8] Zhang Z., Wan M. (2003). Synth. Met..

[cit9] Rajak D. K., Pagar D. D., Kumar R., Pruncu C. I. (2019). J. Mater. Res. Technol..

[cit10] Sazou D. (2001). Synth. Met..

[cit11] Sunderland K., Brunetti P., Spinu L., Fang J., Wang Z., Lu W. (2004). Mater. Lett..

[cit12] Sen T., Mishra S., Shimpi N. G. (2016). RSC Adv..

[cit13] MacDiarmid A. G. (2001). Curr. Appl. Phys..

[cit14] Zhang D., Kang Z., Liu X., Guo J., Yang Y. (2022). Sens. Actuators, B.

[cit15] Vinodh R., Babu R. S., Sambasivam S., Muralee Gopi C. V. V., Alzahmi S., Kim H. J., de Barros A. L. F., Obaidat I. M. (2022). Nanomater.

[cit16] Osuna V., Vega-Rios A., Zaragoza-Contreras E. A., Estrada-Moreno I. A., Dominguez R. B. (2022). Biosens.

[cit17] Hu Y., Hojamberdiev M., Geng D. (2021). J. Mater. Chem. C.

[cit18] Khan S. B., Faisal M., Rahman M. M., Jamal A. (2011). Sci. Total Environ..

[cit19] Jin H., Wang N., Xu L., Hou S. (2010). Mater. Lett..

[cit20] Palard M., Balencie J., Maguer A., Hochepied J. F. (2010). Mater. Chem. Phys..

[cit21] Sun C., Li H., Wang Z. X., Chen L., Huang X. (2004). Chem. Lett..

[cit22] Carrettin S., Concepción P., Corma A., López Nieto J. M., Puntes V. F. (2004). Angew. Chem., Int. Ed..

[cit23] Park S., Vohs J. M., Gorte R. J. (2000). Nature.

[cit24] CUI Q., DONG X., WANG J., LI M. (2008). J. Rare Earths.

[cit25] Tsud N., Skála T., Mašek K., Hanyš P., Takahashi M., Suga H., Mori T., Yoshikawa H., Yoshitake M., Kobayashi K., Matolín V. (2010). Thin Solid Films.

[cit26] Li L., Liu H., Wang Y., Jiang J., Xu F. (2008). J. Colloid Interface Sci..

[cit27] Rahman M. M., Hussein M. A., Alamry K. A., Al-Shehry F. M., Asiri A. M. (2018). Nano-Struct. Nano-Objects.

[cit28] Li T., Wang X., Liu P., Yang B., Diao S., Gao Y. (2019). Synth. Met..

[cit29] Xu G., Wang N., Wei J., Lv L., Zhang J., Chen Z., Xu Q. (2012). Ind. Eng. Chem. Res..

[cit30] Guo B., Zhao Y., Wu W., Meng H., Zou H., Chen J., Chu G. (2013). Chem. Eng. Process..

[cit31] Feng J. X., Tong S. Y., Tong Y. X., Li G. R. (2018). J. Am. Chem. Soc..

[cit32] Ren L., Zhang G., Yan Z., Kang L., Xu H., Shi F., Lei Z., Liu Z. H. (2015). ACS Appl. Mater. Interfaces.

[cit33] Zhang J., Wang J., Yang J., Wang Y., Chan-Park M. B. (2014). ACS Sustainable Chem. Eng..

[cit34] Xu J., Li G., Li L. (2008). Mater. Res. Bull..

[cit35] He Y. (2005). Mater. Chem. Phys..

[cit36] Sasikumar Y., Kumar A. M., Gasem Z. M., Ebenso E. E. (2015). Appl. Surf. Sci..

[cit37] Kuralay F., Dumangöz M., Tunç S. (2015). Talanta.

[cit38] Karimi A., Husain S. W., Hosseini M., Azar P. A., Ganjali M. R. (2018). Sens. Actuators, B.

[cit39] Wu J. (2016). Int. J. Electrochem. Sci..

[cit40] Tiwari I., Gupta M., Sinha P., Aggarwal S. K. (2012). Electrochim. Acta.

[cit41] Bard A. J. and Faulkner L. R., Electrochemical Methods: Fundamentals and Applications, Wiley, 2nd edn, 2000

[cit42] Douglas S. R. C. , Skoog A., West D. M., and James Holler F., Fundamentals of analytical chemistry, Thomson-Brooks/Cole, Belmont, Calif, 9th edn, 2014

[cit43] Adeosun W. A., Asiri A. M., Marwani H. M., Rahman M. M. (2020). ChemistrySelect.

[cit44] Neal C. J., Gupta A., Barkam S., Saraf S., Das S., Cho H. J., Seal S. (2017). Sci. Rep..

[cit45] Pirmohamed T., Dowding J. M., Singh S., Wasserman B., Heckert E., Karakoti A. S., King J. E. S., Seal S., Self W. T. (2010). Chem. Commun..

[cit46] Singh S., Singh M., Mitra K., Singh R., Sen Gupta S. K., Tiwari I., Ray B. (2017). Electrochim. Acta.

[cit47] Alamry K. A., Khan A., Hussein M. A., Alfaifi S. Y. (2022). Microchem. J..

[cit48] Xu Q., Mao C., Liu N. N., Zhu J. J., Shen J. (2006). React. Funct. Polym..

[cit49] Ansari A. A., Sumana G., Khan R., Malhotra B. D. (2009). J. Nanosci. Nanotechnol..

[cit50] Yao H., Li N., Xu S., Xu J. Z., Zhu J. J., Chen H. Y. (2005). Biosens. Bioelectron..

[cit51] Tong Z., Yuan R., Chai Y., Xie Y., Chen S. (2007). J. Biotechnol..

[cit52] Xu X., Tian B., Zhang S., Kong J., Zhao D., Liu B. (2004). Anal. Chim. Acta.

[cit53] Wen Z., Ci S., Li J. (2009). J. Phys. Chem. C.

[cit54] Lu W., Chang G., Luo Y., Liao F., Sun X. (2011). J. Mater. Sci..

[cit55] Hu J., Li F., Wang K., Han D., Zhang Q., Yuan J., Niu L. (2012). Talanta.

[cit56] Zhang L., Li H., Ni Y., Li J., Liao K., Zhao G. (2009). Electrochem. Commun..

